# A simple mouse model of pericardial adhesions

**DOI:** 10.1186/s13019-019-0940-9

**Published:** 2019-06-28

**Authors:** Ai Kojima, Tomohisa Sakaue, Mikio Okazaki, Fumiaki Shikata, Mie Kurata, Yuuki Imai, Hirotomo Nakaoka, Junya Masumoto, Shunji Uchita, Hironori Izutani

**Affiliations:** 1 Department of Cardiovascular and Thoracic Surgery, Ehime University Graduate School of Medicine, Shitsukawa, Toon, Ehime 791-0295 Japan; 2 Department of Cell Growth and Tumor Regulation, Proteo-Science Center (PROS), Shitsukawa, Toon, 791-0295 Ehime Japan; 3 Department of Thoracic, Breast and Endocrinological Surgery, Okayama University Graduate School of Medicine, Dentistry and Pharmaceutical Sciences, 2-5-1 Shikata-cho, Kita-ku, Okayama, 700–8558 Japan; 4 Paediatric Cardiac Surgery, Queensland Children’s Hospital, South Brisbane, QLD Australia; 5 Department of Pathology, Division of Analytical Pathology, Ehime University Graduate School of Medicine, Shitsukawa, Toon, 791-0295 Ehime Japan; 6 Department of Pathology, Proteo-Science Center (PROS), Shitsukawa, Toon, 791-0295 Ehime Japan; 7 Division of Integrative Pathophysiology Proteo-Science Center, Ehime University Graduate School of Medicine, Shitsukawa, Toon, 791-0295 Ehime Japan

**Keywords:** Pericardial adhesions, Mouse model, Talc

## Abstract

**Background:**

Postoperative pericardial adhesions are considered a risk factor for redo cardiac surgery. Several large- and medium-size animal models of pericardial adhesions have been reported, but small animal models for investigating the development of anti-adhesion materials and molecular mechanisms of this condition are lacking. In this study, we aimed to establish a simple mouse model of pericardial adhesions to address this gap.

**Methods:**

We administered blood, minocycline, picibanil, and talc into the murine pericardial cavity via one-shot injection. Micro-computed tomography analyses of contrast agent-injected mice were carried out for methodological evaluation. We investigated various dosages and treatment durations for molecules identified to be inducers of pericardial adhesion. The adhesive grade was quantified by scoring the strength and volume of adhesion tissues at sacrificed time points. Histological staining with hematoxylin and eosin and Masson’s trichrome, and immunostaining for F4/80 or αSMA was performed to investigate the structural features of pericardial adhesions, and pathological features of the pericardial adhesion tissue were compared with human clinical specimens.

**Results:**

Administration of talc resulted in the most extensive pericardial adhesions. Micro-computed tomography imaging data confirmed that accurate injection into the pericardial cavity was achieved. We found the optimal condition for the formation of strong pericardial adhesions to be injection of 2.5 mg/g talc for 2 weeks. Furthermore, histological analysis showed that talc administration led to an invasion of myofibroblasts and macrophages in the pericardial cavity and epicardium, consistent with pathological findings in patients with left ventricular assistive devices.

**Conclusions:**

We successfully established a simple mouse model of talc-induced pericardial adhesions, which mimics human pathology and could contribute to solving the clinical issues related to pericardial adhesions.

## Background

Postoperative pericardial adhesions are frequently seen after cardiac surgery. Strong adhesions under the sternum and around the heart and major vessels make repeat sternotomy complicated, and often result in surgical injuries and perioperative bleeding [1, 2]. Several developmental studies have shown that Seprafilm® (hyaluronic acid-carboxymethylcellulose) and TachoSil® hemostatic sponge are ideal barrier agents to prevent adhesive tissue formation [3, 4]. However, clinical outcomes of these materials have not been satisfactory, suggesting the need for additional in vivo research to elucidate the molecular mechanisms of adhesion development using animal models.

Several animal models which have utility in the development of materials that prevent pericardial adhesions have been reported. For example, a surgical injury model by abrasion with gauze or placement of sutures has been frequently performed in rabbits [5–7], dog [8, 9], and pigs [10]. Injection of talc into the pericardial space has been reported to produce pericardial adhesions in dogs [11] and pigs [12]. While these currently available large- and medium-sized animal models with thoracotomies are useful for the development of adhesion-prevention materials, small animal models are more valuable for investigations of the precise molecular processes of cardiac adhesion due to their ease of handling, lower costs, and increased reproductive capacity compared with larger animals. In this study, we aimed to establish a simple mouse model of pericardial adhesions to identify the molecular mechanisms which lead to adhesion development.

## Materials and methods

### Study design and setting

**Fig. 1:**
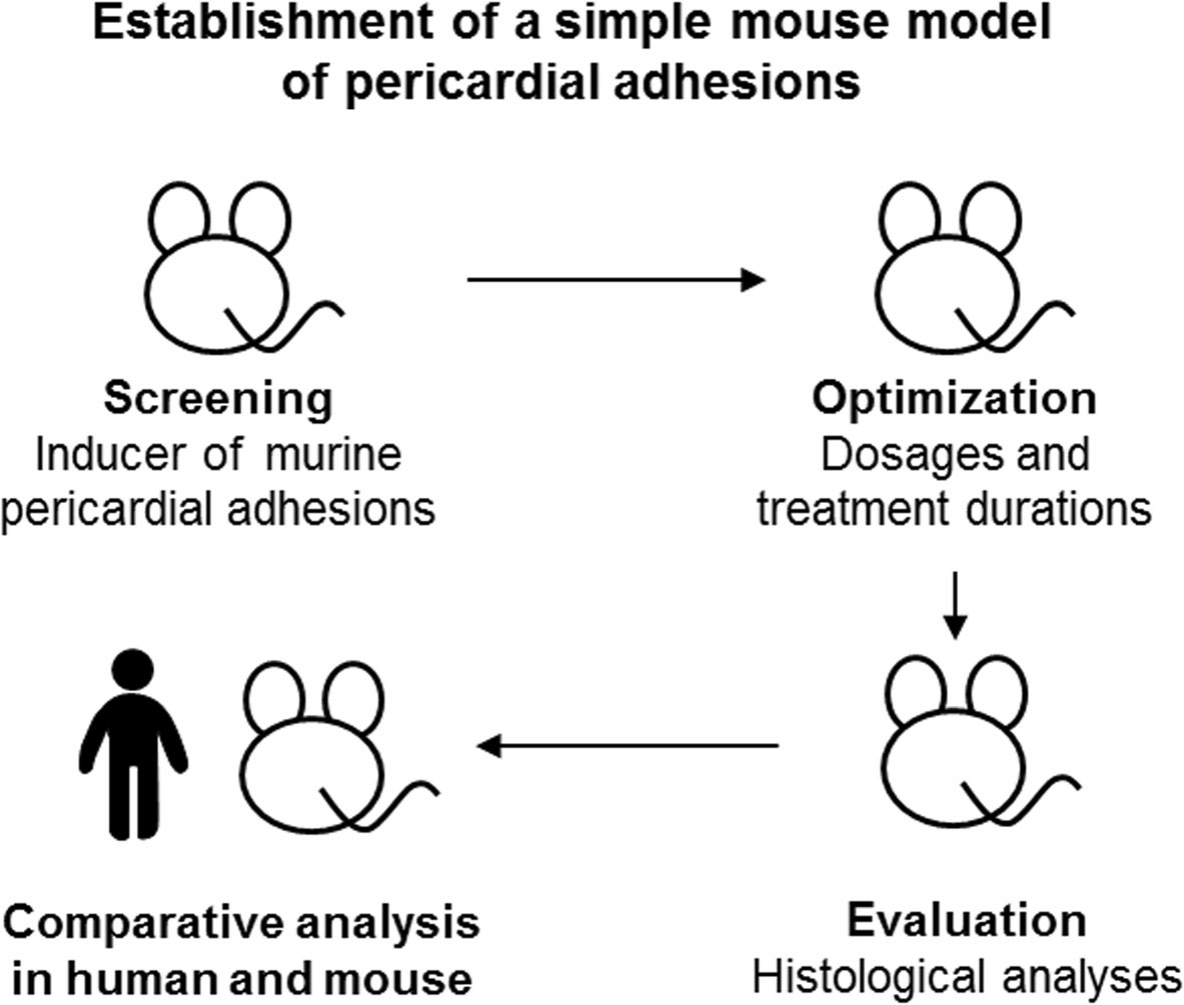
Experimental strategy for establishment of the mouse model of pericardial adhesion. To screen for inducers of pericardial adhesion, several compounds or blood samples were injected into the pericardial cavity using a one-shot injection method. Assessment of the induction of pericardial adhesion was carried out by micro-computed tomography imaging. Optimal condition (dosages and treatment durations) were evaluated, and adhesion tissues were evaluated by histological analysis. Finally, the pericardial adhesion tissues that formed in mice were compared with those of humans by histological analysis

The experimental strategy and study design for establishment of the mouse model of adhesion is shown in Fig. 1.

### Chronic pericardial adhesion model in mice

Eight-week old C57/Black6 (B6) male mice were purchased from CLEA Japan (Tokyo, Japan). Anesthesia was maintained with 2.5% isoflurane in oxygen for all procedures. A small skin incision was made into the abdomen. After cutting the peritoneum and reaching the abdominal cavity, either 500 μL of low- (2.5 mg/g) or high-dose talc (5 mg/g) (Unitalc; Nobelpharma, Tokyo, Japan), 300 μL of minocycline (2 mg/mL; Pfizer, Tokyo, Japan), 375 μL of picibanil (3.0 KE/kg Chugai Pharmaceutical, Tokyo, Japan), 300 μL of heparin-treated blood from donor mice, or saline solution was injected into the pericardial cavity from the diaphragm side using a 23-gauge needle attached to a 1 mL syringe. Injection volumes and doses were determined by referring to published studies [13, 14]. At 1, 2, and 4 weeks after operation, mice were sedated with ketamine (0.1 mg/g) and xylazine (0.01 mg/g) to score the strength of adherence and measure the volume of adhesion tissue.

### Adhesion scoring

Grade of pericardial adhesions were defined by two parameters, as shown in Table 1.

**Table 1:** Grading of pericardial adhesions

Category A: Volume of adhesion tissue	
Grade	Description
0	No adhesion
1	One third of the heart surface area is covered
2	Two thirds of the heart surface area are covered
3	Ninety percent of the heart surface area is covered
4	Entire heart surface area is covered
Category B: Strength of adherence	
Grade	Description
0	No adhesion.
1	Adhesion tissue is easily divided
2	Adhesion tissue is partially divided
3	Adhesion tissue is completely adhered to epicardium

### Micro-computed tomography evaluation

To confirm the accuracy of our technique of injection into the pericardial cavity from the diaphragm side, we performed computed tomography (CT) imaging following pericardial injection of a contrast agent with no adhesion inducer. The contrast agent (Omnipaque; Daiichi-Sankyo Pharmaceutical, Tokyo, Japan) was injected under complete sedation (as described above for adhesion inducers). After mice were euthanized by pentobarbital overdose, the area of the pericardial cavity that was stained by contrast agent was visualized using a micro-CT (μCT) 35 scanner (SCANCO Medical AG, Bruittesellen, Switzerland).

### Histological evaluation

Specimens were stained with hematoxylin and eosin (H&E) and Masson’s trichrome, and immunostained for F4/80 or alpha smooth muscle actin (αSMA), as previously reported [15, 16]. In brief, heart samples were harvested from PBS- or talc-injected mice and fixed with 4% paraformaldehyde overnight. Tissues were then embedded in paraffin and sectioned into 10 μm slices along the cephalocaudal axis. For avidin-biotin complex (ABC) immunostaining, sections were incubated with anti-F4/80 antibody (1/100 dilution; Clone# A3–1, Abcam, Cambridge, MA) or anti-αSMA antibody (1/100 dilution; Clone# SPM332, Novus Biologicals, Littleton, CO, USA) overnight, followed by horseradish peroxidase-labelled secondary antibody (Nichirei Bioscience, Tokyo, Japan) for 1 h. Visualization of F4/80 and αSMA was achieved via 3,3′-diaminobenzidine tetrahydrochloride (DAB) reaction. Images were taken using a microscope (Olympus, Tokyo, Japan).

### Statistical analysis

Quantitative adhesion data were calculated from four independent experiments (*n* = 4 mice per experiment) and are presented as means ± standard errors (SEs). Statistical analyses were carried out using GraphPad software (GraphPad Prism; GraphPad Software, San Diego, CA).

## Results

### Verification of pericardial injection

**Fig. 2:**
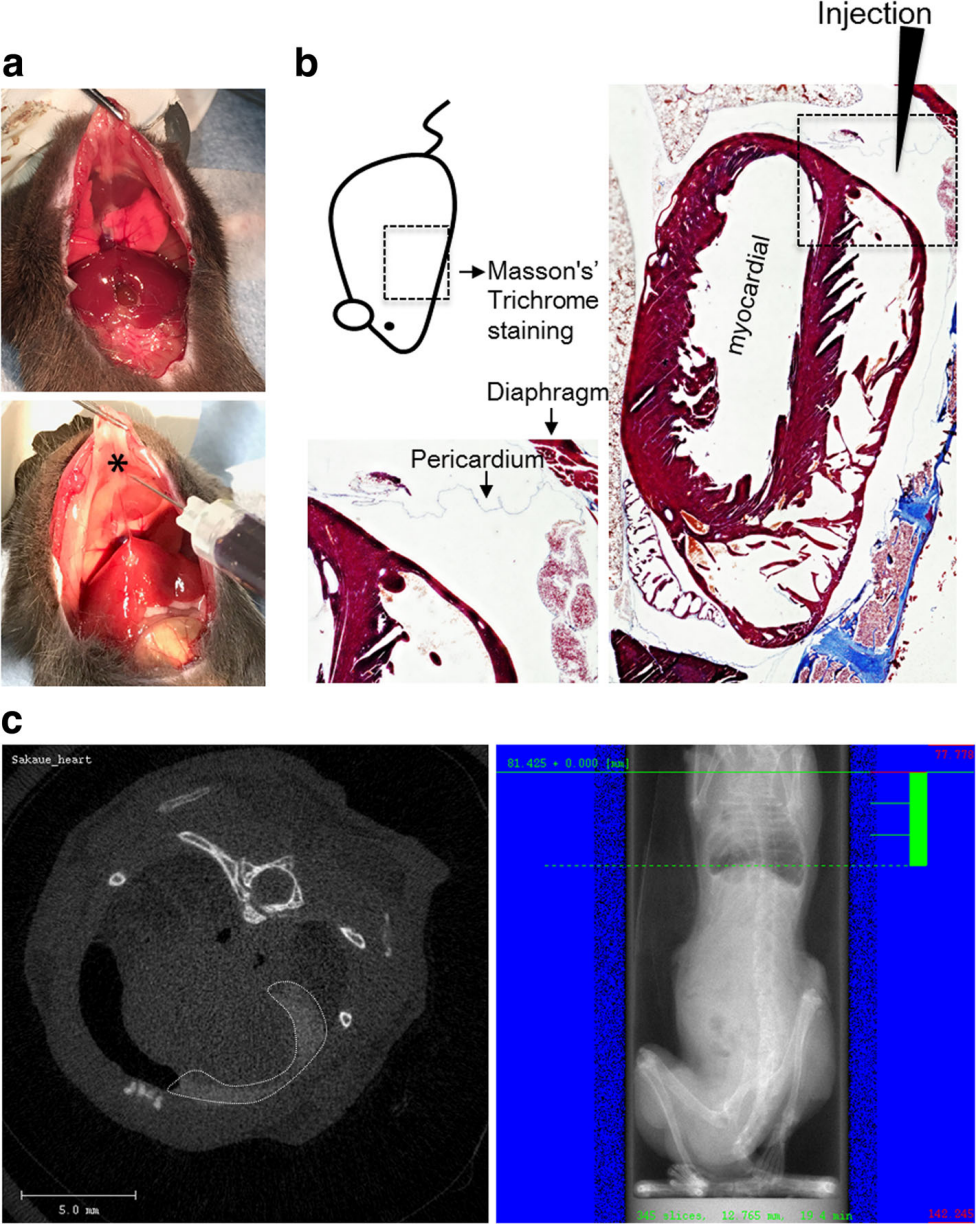
Histological and micro-computed tomography evaluation of the one-shot injection method. **a** Potential adhesion-inducer compounds were injected from the diaphragm side, indicated by the asterisk. **b** Masson’s trichrome staining of the chest, cross section, in a mouse. The lower left area shows the pericardium in a broken line in the right panel. The arrowhead indicates the injection site. **c** Representative chest computed tomography image (left panel) and photographing range (right panel) of a contrast reagent-injected mouse. The broken line in the left panel indicates Omnipaque-derived signals

The success of pericardial injection was verified by histological staining with Masson’s trichrome stain (Fig. 2a). A small space was observed between the epicardial membrane and the pericardium on the diaphragm side, which completely corresponds to the injection site (Fig. 2b). Additionally, Omnipaque-derived CT signals were observed in the pericardial cavity, confirming that contrast agents had been successfully delivered (Fig. 2c).

### Identification of inducer of adhesions in the pericardial cavity

**Fig. 3:**
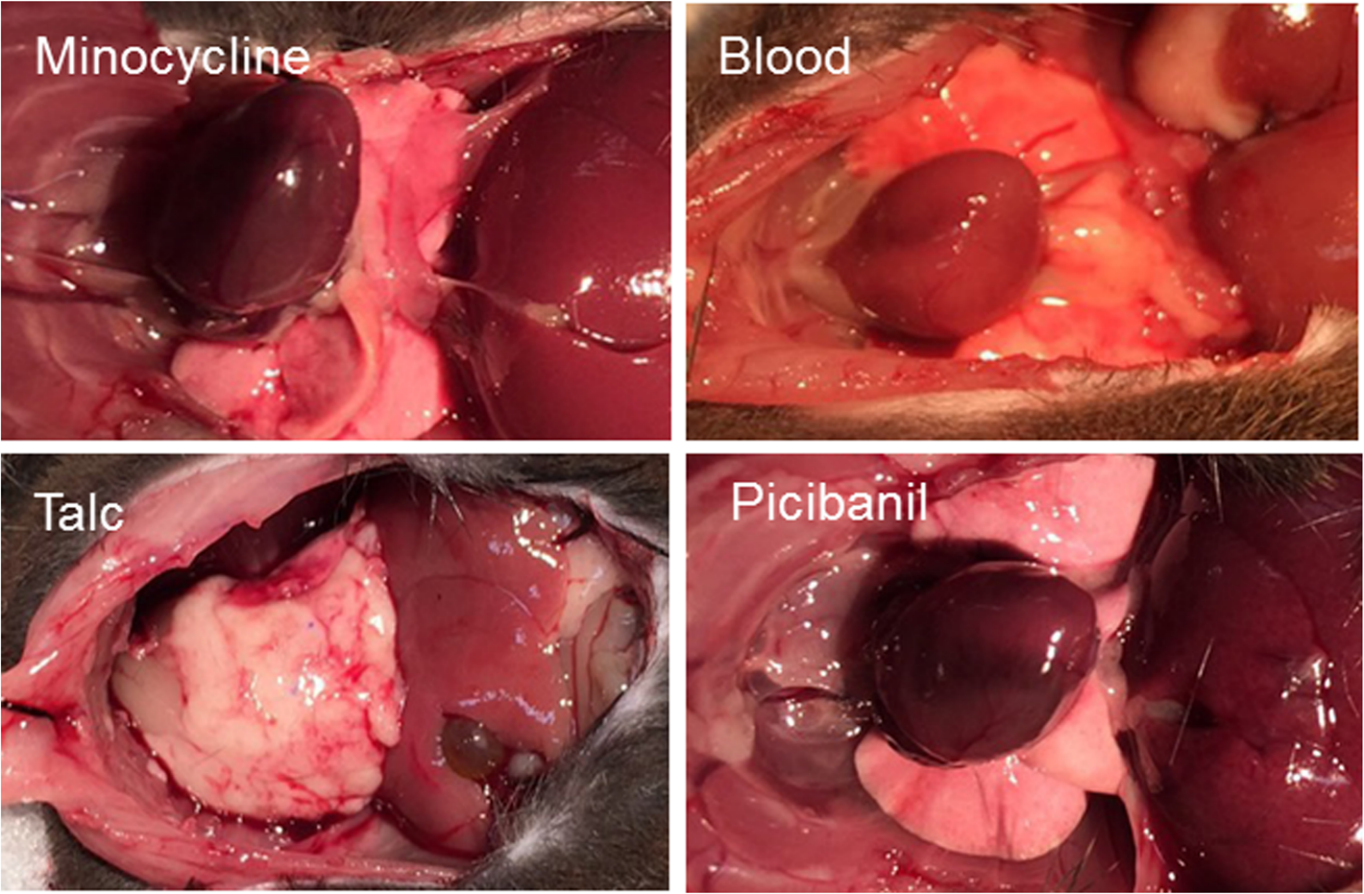
Autopsy photos of minocycline-, blood-, talc- and picibanil-injected mice. We injected 500 μL of talc (5.0 mg/g), 300 μL of minocycline (2 mg/mL) (upper left), 375 μL of picibanil (3 KE/kg) (lower right), or 300 μL of heparin-treated blood (lower left) into the murine pericardial cavity. Two weeks later, the chest was opened under complete sedation, and anatomy images were recorded

The specific chemical compound that was responsible for the formation of pericardial adhesion was identified through the injection of clinically used inducers of adhesion as well as injection of blood, which is assumed to cause adhesion due to bleeding. As shown in Fig. 3, talc-injected mice exhibited strongly induced pericardial adhesions within 2 weeks. The hearts were completely covered by adhesion tissue, while there was no obvious adhesion tissue in the abdominal cavities. Notably, minocycline-, picibanil-, and blood-injected mice did not develop any macroscopic adhesion tissue (Fig. 3). These data indicate that our one-shot injection method was sufficient, and that talc was the most effective inducer of murine pericardial adhesions.

### Optimization of talc-induced pericardial adhesion model

**Fig. 4:**
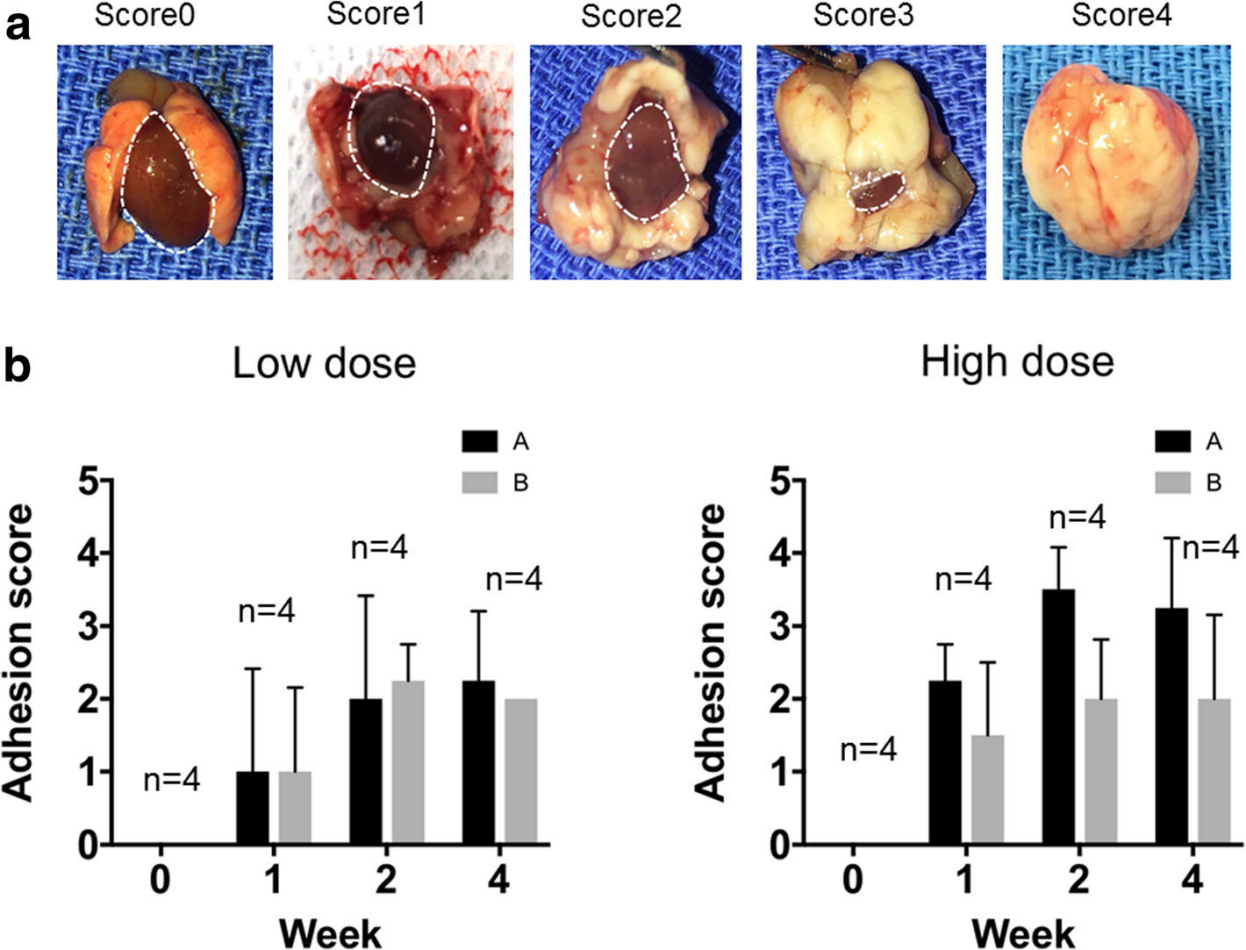
Optimization of the talc-induced pericardial adhesion method. Talc was injected at a low dose (500 μL of 2.5 mg/g) or high dose (500 μL of 5.0 mg/g) into the murine pericardial cavity. After 1, 2, and 4 weeks, the adhesive grade was calculated by scoring the strength and volume of adhesion tissues. **a** Representative heart photos from each scored mouse. The broken line indicates the heart surface area. **b** Relationship of adhesion score and treatment term (by week) following low-dose (left panel, 2.5 mg/g) and high-dose (right panel, 5.0 mg/g) injection of talc (*n* = 4). Categories A and B represent the volume and strength of adhesion tissues, respectively

Representative views of the volume of pericardial adhesions for each score are shown in Fig. 4a. Adhesion tissue was seen in all mice at 1 week after talc injection (Fig. 4b). Although the volume of pericardial adhesions was two-fold higher in the high-dose groups compared with the low-dose groups, there were no differences among the strength of adhesion tissue at any timepoint (Fig. 4b). The scores for volume and strength of adhesion tissue were higher at 2- than 1-week post injection, but no further differences were seen between 2 and 4 weeks, indicating that 2 weeks was sufficient to induce maximal adhesions (Fig. 4b). These results indicated that treatment with 2.5 mg/g of talc for 2 weeks was the optimal method to produce pericardial adhesions in mice.

### Histological analysis

**Fig. 5:**
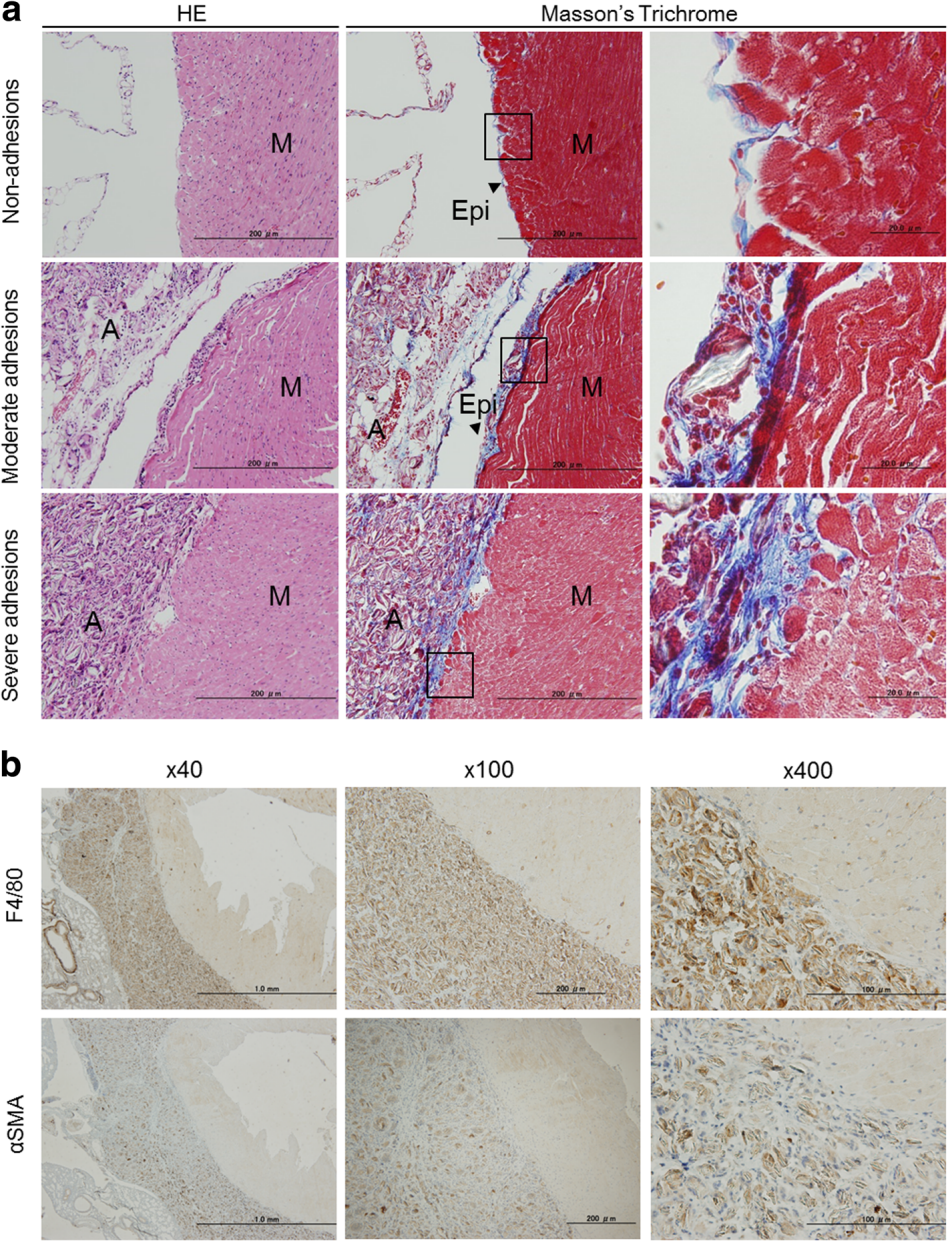
Histological analysis of pericardial adhesion tissues. a Hematoxylin and eosin staining (left panels) and Masson’s trichrome staining (middle and right panels) of non-adhesions, moderate adhesions (Grade 2 in category B) and severe adhesions (Grade 3 in category B). The right-hand panels show enlarged epicardia in areas corresponding to the lines in the middle panels. **b** Immunohistochemical staining of F4/80 (upper panels) and alpha smooth muscle actin (lower panels)

Masson’s trichrome and H&E staining were performed to clarify the histological differences of adhesion strength between adhesion strength scores of 0, 2, and 3. Crystals of talc and elastic fibers within adhesion tissues were stained blue by Masson’s trichrome and were observed in both moderate adhesions (category B2) and severe adhesions (category B3) (Fig. 5a). A strong inflammatory reaction appeared on the epicardium and myocardium in cases of severe adhesion (category B3) (Fig. 5a). Immunohistochemical staining showed that αSMA-positive myofibroblasts and F4/80-positive macrophages had strongly invaded the adhesion tissue (Fig. 5b). These data suggested that talc-induced inflammation might contribute to the formation of pericardial adhesions with invasion of immune cells.

**Fig. 6:**
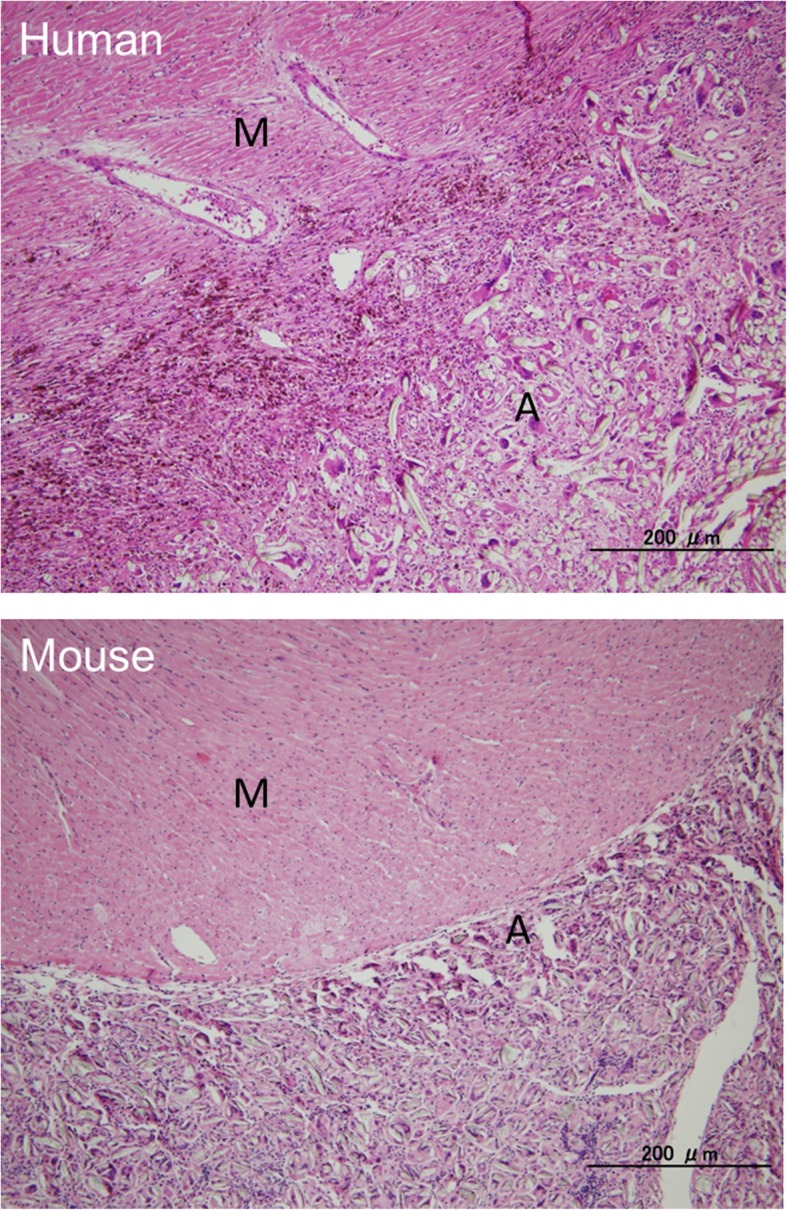
Comparative analysis of histology in human and mice pericardial adhesion tissue. Hematoxylin and eosin staining was performed to compare human and mouse adhesion tissues from the pericardium. The upper panel shows human heart specimens taken from autopsy patients with left ventricular assist devices. The lower panel shows murine heart samples treated with injection of 500 μL of talc (2.5 mg/g) for two weeks into the pericardial cavity

Invasion of macrophages and myofibroblasts, along with deposition of elastic fibers and erosion of the myocardium, were commonly observed in both murine and human pericardial adhesion tissue (Fig. 6). These data demonstrate that our mouse model is representative of foreign-body-induced pericardial adhesions in humans, and could be useful for elucidating the molecular mechanism of these adhesions.

## Discussion

Although pericardial adhesions are a common consequence of cardiac surgery, medical and surgical therapies have not yet been established because the molecular mechanism by which adhesions develop is unclear. Previous investigators have reported several large- and middle-sized animal models of pericardial adhesions such as rabbits [5–7], pigs [10], and dogs [8, 9], which have been used to describe materials that prevent the formation of adhesion tissue. These studies have limited translation to human studies and the procedures involved often require the support of a ventilators and advanced surgical techniques, making them expensive and difficult to reproduce on larger scales. Despite these limitations, there have been no small-animal studies, which are better suited for investigation of genetic and molecular biology. To the best of our knowledge, the present study is the first description of a mouse model of pericardial adhesions without sternotomy and thoracotomy. Through the use of a simple model, we evaluated the injection method, adhesion-promoting compounds, dosage, and time course for optimal for adhesion formation.

Nakatani et al. reported that the murine pericardium has numerous pores, unlike that of humans [17], which directly connect the pericardium to the pleural cavity. Fukuo et al. also showed that particulate matter can pass from the pleural cavity into the pericardial cavity [18]. Based on this evidence, we traced injected samples into the pericardial cavity using a μCT device. Our CT imaging data confirmed that the injected contrast reagents flowed into the pericardial cavity. Furthermore, talc-induced adhesion tissue was not observed in the pleural cavity. These data suggest that diffusion of injected materials through pericardial pores is limited by particle size.

Generally, talc, minocycline, and picibanil are known to be inducers of pleurodesis in patients with pneumothorax and malignant pleural effusion. All three compounds stimulate immune cells, with subsequent enhancement of inflammatory reactions. Several researchers have demonstrated, through experimental and clinical studies, that minocycline induces pericardial adhesions in dogs and humans [19–21] through injury of the epicardial-pericardial surface by acidic stimulation. This indirectly indicates that talc, minocycline, and picibanil might be able to induce adhesions in the murine pericardium. Unexpectedly, we found that talc was the best inducer of adhesion in the pericardial cavity, and no macroscopic adhesion tissue was obvious in minocycline-, picibanil- or blood-injected mice for 2 weeks post injection. Similar results have been reported for a porcine model [22]. Taken together, the data from our study and others suggest that mice and pigs might possess strong resistant properties to the formation of pericardial adhesions by minocycline, picibanil, or bleeding, which is inconsistent with humans.

Strong pericardial adhesions are a significant cause of morbidity and mortality due to surgical injuries and severe bleeding related to complicated technical aspects of reoperations [23]. In order to reproduce such clinical issues in mice, we investigated the treatment term and dosage of talc, and identified optimal conditions for establishing adhesions. Histology showed that fibrosis was ectopically formed within the pericardial cavity. Similar findings were observed in heart specimens taken from patients with left ventricular assist devices. Cannata et al. also showed that the connective tissue of adhesions fills the space between the pericardial layers in humans [24]. Our data and previous evidence indicate that the pathology of our mouse model is similar to that of humans, justifying its utility for elucidating the molecular mechanisms of pericardial adhesions.

In recent years, genome editing technologies have significant increased our understanding of pathological and physiological events [25]. In the future, combination of our mouse model with the latest genome-editing technologies could contribute to clarification of the entire molecular mechanism of pericardial adhesion, which could inform the development of human therapies.

## Conclusion

In the present study, we successfully established a simple mouse model of pericardial adhesions by one-shot injection of talc into the murine pericardial cavity. Our histological data demonstrate that talc-induced immune reactions are effective for the induction of adhesions between the pericardium and heart, which are structurally similar to those of patients with left ventricular assist devices. We believe that our animal model can contribute to solving the clinical issues related to pericardial adhesions.

## Data Availability

All data are included in this article.

## Abbreviations

ABC: Avidin-biotin complex
B6: Black 6 mice
CT: Computed tomography
DAB: 3,3'′’-diaminobenzidine tetrahydrochloride
H&E: Hematoxylin and eosin
SE: Standard error
αSMA: Alpha smooth muscle actin
